# Genus *Tinospora*: Ethnopharmacology, Phytochemistry, and Pharmacology

**DOI:** 10.1155/2016/9232593

**Published:** 2016-08-28

**Authors:** Sensen Chi, Gaimei She, Dan Han, Weihua Wang, Zhao Liu, Bin Liu

**Affiliations:** ^1^School of Chinese Pharmacy, Beijing University of Chinese Medicine, Beijing 100102, China; ^2^National Institute of Metrology, Beijing 100013, China

## Abstract

The genus* Tinospora* includes 34 species, in which several herbs were used as traditional medicines by indigenous groups throughout the tropical and subtropical parts of Asia, Africa, and Australia. The extensive literature survey revealed* Tinospora* species to be a group of important medicinal plants used for the ethnomedical treatment of colds, headaches, pharyngitis, fever, diarrhea, oral ulcer, diabetes, digestive disorder, and rheumatoid arthritis. Indian ethnopharmacological data points to the therapeutic potential of the* T*.* cordifolia* for the treatment of diabetic conditions. While* Tinospora* species are confusing in individual ingredients and their mechanisms of action, the ethnopharmacological history of those plants indicated that they exhibit antidiabetic, antioxidation, antitumor, anti-inflammation, antimicrobial, antiosteoporosis, and immunostimulation activities. While the clinical applications in modern medicine are lacking convincing evidence and support, this review is aimed at summarizing the current knowledge of the traditional uses, phytochemistry, biological activities, and toxicities of the genus* Tinospora* to reveal its therapeutic potentials and gaps, offering opportunities for future researches.

## 1. Introduction

The genus* Tinospora* (Menispermaceae), 34 species included, is a large glabrous deciduous climbing shrub [1]. The species have been widely distributed throughout the tropical and subtropical parts of Asia, Africa, and Australia. At present, 6 species and 2 varieties are found in China, in which* T*.* hainanensis* and* T*.* guangxiensis* Lo. as endemic species are mainly distributed in Guangxi and Hainan.* T*.* sinensis* and* T*.* capillipes* are officially listed in the 2015 edition of the* Chinese Pharmacopoeia* (中华人*民共*和*国药*典) with the Chinese names “Kuan Jin Teng” (*宽筋藤*) and “Jin Guo Lan” (金果榄), respectively [2]. Those herbs in genus* Tinospora* are mostly heat-clearing and detoxifying (清热解毒). And they are commonly used as domestic folk medicine for the treatment of colds, headaches, pharyngitis, fever, diarrhea, oral ulcer, digestive disorder, and rheumatoid arthritis. For instance, Indian Ayurveda usually employs* T*.* cordifolia* in treatment for diabetes. Its obvious antidiabetic properties attract modern researches' attention [3].* T*.* sinensis* is the major composition in a famous traditional formula “Si Wei Zang Mu Xiang” powder (四*味藏*木香), used for the treatment of wind-heat disease (风热病) and deficiency-heat syndrome (虚热证) [3, 4]. This family is a rich source of terpenes and alkaloids; the ongoing exploration for bioactive constituents also revealed the presence of C_6_-C_3_ derivatives, polysaccharides, and other components with exact structures. Modern pharmacological researches and clinical practices demonstrated that* Tinospora* species possess a wide spectrum of activities, including antidiabetic [3], antioxidation [5, 6], antitumor [7, 8], anti-inflammation [9, 10], antimicrobial [11], antiosteoporosis [12], and immunostimulation effect [13]. In clinic, genus* Tinospora* is commonly used to protect and support the immune system, to prevent upper respiratory infections, to lower oral ulcer, to treat diabetes, to be an adjunctive therapy in cancer, and to protect the liver. However, a systematic review of these studies has not been performed to date. This paper summarized the research achievements of the medicine plants in genus* Tinospora* ranging from clinical studies to phytochemistry and pharmacology areas. It is expected that such a paper would on one hand serve as an available database for future researches and on the other hand enable experts in the field to determine if the research on genus* Tinospora *is on proper path.

## 2. Traditional Uses and Ethnopharmacology

Those species in this genus have traditionally been used as therapeutic remedies (Table 1). On the basis of our investigations, they are clinically applied for the treatment of fever and parasitic diseases, mouth, skin, respiratory, and urinary tract infections, oral ulcer, and diabetes, in addition to being an adjunctive therapy in cancer and to protecting the liver [14–22]. The official record about species in China of genus* Tinospora* appeared in “*Bencao Gangmu Shiyi*” [Zhao Xuemin, Qing dynasty, year 1765,* Bencao Gangmu Shiyi* (本*草纲*目*拾遗*)]. It is recorded that* T*.* capillipes* is primarily used on the therapy of dysentery, diarrhea, and abdominal pain with heat, toxic furuncle ulcer and sore throat. It is also the traditional medicine of* Mao*,* Dong,* and* Yao* minority of China, being applied to the treatment of acute laryngopharyngitis, tonsillitis, gastroenteritis, and bacillary dysentery [23, 24]. Earlier, “*Jingzhu Bencao*” [Dimaer·Danzeng PengCuo, Qing dynasty, year 1745,* Jingzhu Bencao* (*晶珠*本草)], which is an ancient Chinese Tibetan medicinal encyclopedia, recorded that* T*.* sinensis* has been used for relieving rigidity of muscle and activating collaterals (*舒筋*活络) and alleviating pain (活血止痛). Its tropism of taste is Xin and Ku and in the liver channel (味辛, 苦, 主肝经). Chinese Dai Medicine (傣医药学) claimed that* T*.* sinensis* could also regulate and invigorate the blood (*调补*气血) and tranquilize the mind (镇心安神). It is clinically used to treat palpitation (心悸) and improve the frail physique (*体弱无力*). Thailand Lanna Medicine (泰国兰纳医药学) claimed that* T*.* sinensis* was more inclined to clear the heat and cool off the fire (清热降火) inside the body, thus usually clinically applied to relieve high fever and ameliorate the symptoms of diabetes [15–26]. In India,* T*.* cordifolia* referred to as Guduchi (plant which protects from diseases, Sanskrit) has been described in ancient text books of Ayurveda including* Sushrut Samhita* and* Charak Samhita* [27]. According to Ayurveda,* T*.* cordifolia* itself possesses a bitter, pungent, and astringent taste. The bitter taste is said to improve metabolic activity, even at a cellular level. It is documented to treat gastrointestinal diseases including dyspepsia, flatulence, gastritis, jaundice, diarrhea, splenomegaly, and hemorrhoids. It has a role in the treatment of metabolic disorders such as diabetes and kidney disorders, and it is currently more inclined to be a research focus. It is prescribed for intermittent fevers, infective conditions, urinary disorders, skin diseases, and eye diseases. In combination with other herbs, it is commonly used as an ingredient to treat gout and rheumatoid arthritis. And the well-ground whole plant is applied to fractures. Moreover,* T*.* cordifolia* shows healthy effects due to its various nutritious compounds and is considered as a general tonic [27, 28]. The diverse, significant ethnomedicinal properties possessed by this genus could be the basis for further researches to investigate the phytochemical and pharmacological aspects of this genus.

## 3. Pharmacological Activities

Scientific studies all over the world for over 50 years have revealed much insight into the pharmacological functions of* Tinospora*. Crude extracts and pure compounds from* Tinospora* plants have shown significant antidiabetic, antioxidant, antitumor, anti-inflammatory, antimicrobial, antiosteoporosis, and immunostimulation properties. In this section, biological activities of the active compounds and mixtures on the species are highlighted.

### 3.1. Antioxidant Activity

Natural plants used in traditional Chinese medicine have antioxidant effects through different mechanisms, including DPPH radical scavenging assay, superoxide anion scavenging assay, hydroxyl radical scavenging assay, and ABTS radical scavenging method [35]. On a comparative basis,* T*.* sinensis* and* T*.* cordifolia* were tested to have equal antioxidant activities [36]. Ilaiyaraja and Khanum evaluated the antioxidant potential of different solvent extracts of leaf and stem of* T*.* cordifolia*. Scavenging effects on DPPH, ABTS radical, hydroxyl radical, and ferric reducing antioxidant power (FRAP) were found to be highest in methanolic extract of leaf and ethyl acetate extract of stem. It is because the polarity of solvent showed a greater effect on the ordered structure of the active phenolic components [5]. The study on leaf and stem powders of* T*.* cordifolia* followed by DPPH method found that the leaf extract powder has higher retention of antioxidant activity than the stem's [37]. As early as 1998, Cavin et al. isolated and identified three compounds N-*cis*-feruloyltyramine, N-*trans*-feruloyltyramine, and secoisolariciresinol from* T*.* crispa*. These ingredients proved to be more active than the synthetic antioxidant butylhydroxytoluene (BHT) [38]. Another investigation showed that the essential oil obtained from leaves of* T*.* cordifolia *demonstrated strong DPPH radical scavenging activity with IC_50_ values of 25 ± 0.3 *μ*g/mL. The components identified in essential oil include alcohols (32.1%), phenols (16.6%), aldehydes (16.2%), fatty acids (15.7%), alkanes (8.3%), esters (3.2%), terpenes (1.2%), and others (4.8%) [39]. The work carried on hitherto rarely studied fruit of* T*.* cordifolia* showed that it is also rich in antioxidant properties with the IC_50_ being 468 *μ*g/g and EC_50_ being 1,472 *μ*g/g, yet the effect of the fruit is not comparable to the other parts [5, 6]. These active components and mixtures could serve as valuable indications to develop them as possible antioxidant drugs for the treatment and the prevention of diseases. More importantly, oxidative stress is proved to be critical for the pathogenesis of diabetes mellitus. Clarification of antioxidant issues could help to understand the treatment of diabetes mellitus and promote the development of antidiabetic natural products [40].

### 3.2. Antidiabetic Activity

Many pharmacologic studies have clearly confirmed the antidiabetic effects of genus* Tinospora in vivo*. Compared with other commonly seen antidiabetic drugs,* T*.* crispa* and* T*.* cordifolia* exhibit more powerful antidiabetic activity, and they have been widely used in Asia and Africa as a remedy mainly in regard to type 2 diabetes mellitus [41]. Ruan et al. explored the hypoglycemic effects of borapetoside A mediated through both the insulin-dependent and the insulin-independent pathways. They found that borapetoside A was able to increase the glucose utilization in peripheral tissues, to reduce the hepatic gluconeogenesis, and to activate the insulin signaling pathway. Comparison of the structures of three borapetosides from* T*.* crispa* clearly suggested that the C-8 stereochemistry plays a key role in hypoglycemic effect, since the active borapetosides A and C possess 8R-chirality while the inactive borapetoside B possesses 8S-chirality. The location of glycoside at C-3 for borapetoside A (while it is C-6 for borapetoside C) and the formation of lactone between C-4 and C-6 for borapetoside A could account for the different potency in hypoglycemic action for these two compounds [42, 43]. Borapetol B is another antidiabetic active property from* T*.* crispa*. By evaluating the blood glucose levels and stimulation of insulin secretion in normoglycemic control Wistar and diabetic Goto-Kakizaki rats, Lokman et al. provided evidence that oral administration of borapetol B has antidiabetic properties mainly due to its stimulation of insulin release [42, 44]. Antidiabetic potential of* T*.* cordifolia* stem is also well proven [3, 45, 46]. Patel and Mishra chose sucrose and maltose as substrates to study the enzyme kinetics and the respective Michaelis-Menten constant; meanwhile maximal velocity values were estimated. Three isoquinoline alkaloids from* T*.* cordifolia*, namely, jatrorrhizine, palmatine, and magnoflorine, showed IC_50_ value as sucrase inhibitor to be 36.25, 23.46, and 9.8 *μ*g/mL and maltase inhibitor to be 22.05, 38.42, and 7.60 *μ*g/mL. Furthermore, the increase in plasma glucose level in* in vivo* studies conducted on rats was significantly suppressed (*P* < 0.01) by all the three alkaloids at 20 mg/kg b.wt. [47]. Kannadhasan and Venkataraman reported that the presence of antioxidant potentials in extract of* T*.* cordifolia* was a potent healer in ameliorating diabetes like tissue damage* in vivo *[48]. It is perfectly consistent with the report that* T*.* cordifolia* has a significant (*P* < 0.05) effect in ameliorating the specific parameters toward normal in diabetic animals, and it has a level of efficacy that is considerably good compared to standard drug insulin [49]. These experimental results further enrich previous understanding of the antidiabetic activity of genus* Tinospora* and provide scientific data of therapeutic efficacy against diabetes mellitus.

### 3.3. Immunostimulatory Activity

Several* Tinospora* species have effects on humoral immunity, cellular immunity, and nonspecific immunity. Polysaccharide, a kind of natural macromolecule, has widespread occurrence in biological bodies and has many kinds of biological activities, which plays an important role in regulating immune system [50]. Polysaccharides in* Tinospora* represent a structurally diverse class of macromolecules and this structural variability can profoundly affect their cell-type specificity and their biological activity in B cells. Previous studies documented that phytogenic polysaccharides could regulate animals' immune system by combining to membrane receptors of immune cells and initiating the special signaling pathways. This includes stimulating the secretion or proliferation of macrophages, T/B lymphocytes, and natural killer (NK) cells, modulating the release of cytokines, promoting the production of antibodies, and activating the complement system [51]. (1,4)-*α*-D-Glucan (*α*-DG) is a novel immune stimulatory drug from* T*.* cordifolia*. It exhibited macrophages activating abilities through TLR6 signaling and NF-*κ*B activation mechanism, followed by the production of cytokines and chemokines. It can also differentially modulate cytokine production in the spleen and lung on endotoxaemic juvenile rats. In the lung, *α*-DG treatment reduced anti-inflammatory cytokine concentrations of IL-1*β* by 30%, IL-6 by 43%, IFN-*γ* by 46%, and IL-10 by 31% compared to endotoxaemia. In the spleen, *α*-DG treatment decreased the ratio of IL-1*β* to IL-10 by 55% [52–54]. Further data showed that *α*-DG led to significant tachycardia without causing hypotension. Accompanied by significant reduction in the blood hemoglobin and hematocrit concentrations, the effect did not change respiratory variable and/or plasma concentration of inflammatory cytokines in anaesthetized rats [55]. G1-4A, an arabinogalactan polysaccharide from* T*.* cordifolia*, is a well-characterized immunomodulatory in both* in vitro* and* in vivo* systems. Administration (12.5 mg/kg body weight) of G1-4A to mice led to splenomegaly and an increase in the numbers of T cells, B cells, and macrophages. This increase in spleen cellularity was due to* in vivo* proliferation of lymphocytes and upregulation of antiapoptotic genes. G1-4A induced proliferation of B cells was completely inhibited by PI3K inhibitor Ly294002, mTOR inhibitor rapamycin, and NF-*κ*B inhibitor plumbagin. Meanwhile, Akt, ERK, and JNK were activated by G1-4A which finally resulted in the activation of IKK, degradation of I*κ*B-*α*, and translocation of NF-*κ*B to the nucleus [56–58]. In the humoral and cell-mediated immune response,* T*.* cordifolia* preparation enhanced mean hemagglutination antibody titre and reactive oxygen level against various pathogens [59–61]. Polar fractions of* T*.* cordifolia* stem at a dose of 40 mg/kg body weight promoted the nonspecific host and defence of the immune system directly acted on peritoneal macrophages [13].* T*.* crispa* crude extract significantly stimulated RAW 264.7 cell viability (*P* ≤ 0.05) and intracellular INF-*γ*, IL-6, and IL-8 expressions. Study of LC-MS showed that the immunomodulatory active compounds are cordioside, quercetin, eicosenoic acid, and boldine [62]. Other reports indicated that it is the synergistic effect of immunomodulatory active compounds in genus* Tinospora* that contributed to the immunomodulatory activity [61, 63]. As mentioned above, polysaccharides in genus* Tinospora* play a key role in the immunostimulatory activity. The characteristics of polysaccharides, which include backbone structure, branching degree, molecular weight, and three spiral structures, affected the activity of immunity. By activating the inherent and acquired immune system to produce antitumor immunity, the immunomodulatory activity could also contribute to the tumor immunotherapy [64].

### 3.4. Antitumor Activity

Feature chemical components of* Tinospora*, clerodane diterpenes, showed apparent cytotoxic activity against tumor cells [65]. Nowadays, the antitumor effect of the* Tinospora* species has been widely studied* in vivo* and* in vitro*. The mechanisms of the antitumor activity of* Tinospora* species are mainly concentrated on the cytotoxicity and cell apoptosis induced. Epoxy clerodane diterpenes were evaluated for the activity against diethylnitrosamine-induced hepatocyte carcinoma; the results concluded that they kick in blocking carcinogen metabolic activation and enhancing carcinogen detoxification [66, 67]. Aqueous ethanolic ingredients of* T*.* cordifolia* reduced cell proliferation in dose-dependent manner and induced differentiation in C_6_ glioma cells. The cell proliferation inhibition was in part mediated through accumulation in S phase and G2/M arrest, with concomitant suppression of p21 expression and inhibition of cyclin-dependent kinase activity [68]. Treatment of hexane extract fraction of* T*.* cordifolia* on Ehrlich ascites tumor bearing animals resulted in growth inhibition and induction of apoptosis in a dose-dependent manner* in vivo*. The mechanism of action was caspase-3 dependent which caused a nuclear localization of apoptosis, meanwhile the formation of apoptotic bodies, nuclear condensation, typical DNA ladder, and decreased cell number and ascites volume [7]. More and Pai observed that treatment with* T*.* cordifolia* and LPS of macrophage cell line J774A.1 followed the enhanced NADH-oxidase, NADPH-oxidase, and myeloperoxidase production as compared to medium alone [69]. According to recent studies, alkaloids chemical constituents from plants have the characteristics of better antitumor activity, high effect, good tolerance, and slight side effect [70]. Alkaloid especially palmatine from* T*.* cordifolia* significantly decreased tumor size, number and the activity of serum enzyme in DMBA induced skin cancer mice. The alkaloid could also contribute to the reduction of glutathione (GSH), superoxide dismutase (SOD), and catalase; however, it increased DNA damage [71]. The activation of macrophages is another pathway for antitumor activity. (1,4)-*α*-D-Glucan and G1-4A are immunological active polysaccharides from* T*.* cordifolia*. Treatment with G1-4A on a murine lymphoma model increased T cell allostimulatory activity and secretion of IL-12 and TNF*α* by bone marrow derived dendritic cells, leading to the lysis of target tumor cells* in vitro*. Polysaccharides can also activate macrophages, NK cells, APDs, and T and B lymphocytes to enhance antitumor immunity concerned with secretion of cytokines, activation of the complement system, induction of apoptosis of tumor cells, and intracellular signal transduction so as to achieve the tumor inhibitory effect by regulations of the immune system [56, 72]. Sonaimuthu et al. identified taxol producing endophytic fungus* F*.* culmorum* SVJM072 from medicinal plant of* T*.* cordifolia*. Further research showed that the fungal taxol had strong activity against human cancer cell lines by MTT assay and* M. tuberculosis* H37Rv by Radiometric Bactec 460 assay, which could serve as an excellent alternative source and a genetically engineered species for anticancer compounds [73].

### 3.5. Anti-Inflammatory and Antimicrobial Activity

Inflammation is a pathologic condition that includes a wide range of diseases such as rheumatic and immune-mediated conditions, diabetes, and cardiovascular accident. If the process of inflammatory response cannot end normally when cell debris and pathogens were cleared, the biological defence response will become causative factor. Medicinal plants and their secondary metabolites are progressively used in the treatment of diseases as a complementary medicine [74, 75]. Palmatine and jatrorrhizine, mainly separated from tubers of* T*.* capillipes *and* T*.* cordifolia*, have widely been applied in clinic owing to their comparatively broad spectrum inhibitory activity. With the EC_50_ values ranged from 0.0348 to 0.8356 g/L and 0.0240 to 0.8649 g/L, respectively, palmatine and jatrorrhizine exhibited inhibition against many plant pathogens. The two alkaloids vary only in the substituents of position 3 of A ring in terms of the chemical structure. The effectiveness of protoberberine was related to the ammonium N linked at position 7 of aromatic ring in the chemical structure and the inhibitory activity of protoberberine alkaloids altered by the different substituents in A and D rings [76]. The ethanol extract of* T*.* smilacina* showed inhibitory activities on COX-1, COX-2, 5-LO, and PA_2_ with the IC_50_ values of 63.5, 81.2, 92.1, and 30.5 *μ*g/mL, respectively [9]. Considerable decrease in the levels of iNOS, COX-2, and ICAM-1 resulted in the reduced release of proinflammatory mediators like TNF-*α*, IL-4, NO, and IgE [10]. An insight into the antimicrobial activity of the ethanolic extracts of* T*.* cordifolia* indicated that the secondary metabolites exhibited high inhibitory activity against clinically isolated* S*.* aureus* and* K*.* pneumoniae* [11]. It is noteworthy that induction of embryogenic callus of* T*.* cordifolia *with the presence of additional compounds was able to inhibit both Gram-positive and Gram-negative organisms [77]. All in all, inflammation is a complex and multifaceted problem, so the treatment of inflammation is not a one-dimensional remedy. The anti-inflammation of herbal medicine belonging to genus* Tinospora* has a favorable applied perspective; with the help of modification in structure it may assist in reaching a multidimensional therapeutic approach to inflammation in the future [78].

### 3.6. Antiosteoporosis Activity

During recent years, researches in traditional Chinese medicine for the treatment of osteoporosis are increasing, especially in the research and therapeutic application using compound medicine and external medicine, and some progress has been made [79].* T*.* capillipes *and* T*.* cordifolia* were reported to induce alkaline phosphatase activity in mesenchymal stem cells based on ecdysone compounds. At the dosage of 25 *μ*g/mL, ethanolic extracts of* T*.* cordifolia* stimulated the growth of osteoblasts, increased the differentiation of cells into osteoblastic lineage, and increased the mineralization of bone-like matrix on both the osteoblast model systems. Previously, Gao et al. noted that the stem extracts of* T*.* cordifolia* in ovariectomized rats significantly prevented the bone loss in rats induced by ovariectomy without causing proliferative effects in uterus and mammary gland [80, 81]. In addition, the presence of high concentration of calcium in* T*.* cordifolia* made it a natural cure for osteoporosis [12]. Moreover, single or synergistic formulations of* T*.* cordifolia* with* Zingiber officinale* have been used in ancient India medicinal system to treat rheumatoid arthritis [34]. However, the morbidity of osteoporosis has been linked to several factors such as age, gender, and races. In order to get a good outcome of osteoporosis treatment, both surgical management and pharmacological treatment are necessary.

### 3.7. Other Therapeutic Activities

What is more,* Tinospora* has been investigated to exert other pharmacological activities. Cordifolioside A, mainly isolated in* T*.* cordifolia*, has a potential* in vivo* radioprotective effect and* in vitro* cytoprotective activity [82]. The impressive radioprotective efficacy may be related to the attenuation of radiation induced decrease of adherence and spreading, increase of IL-1*β* and GM-CSF levels, and reduction of apoptosis [83]. The radiosensitization by* T*.* cordifolia* may be also due to depletion of GSH and GST, accompanied by elevated levels of LPx and DNA damage of tumor cells [84].

Ethanolic extract of* T*.* sinensis* exhibited an appreciable activity against promastigotes (IC_50_  37.6 ± 6.2 *μ*g/mL) and intracellular amastigotes (IC_50_  29.8 ± 3.4 *μ*g/mL). In hamsters, it resulted in 76.2 ± 9.2% inhibition at 500 mg/kg/day × 5 oral dose levels [19–24].* T*.* cordifolia* (100 mg/kg b.wt. for 15 days daily) used in combination with cisplatin in* L*.* donovani* infected BALB/c mice selectively induced Th1 type of immune response. It was concluded by the enhanced levels of IFN-c and IL-2 with moderate decline of Th2 specific cytokines IL-4 and IL-10 [85].

The least polar alkaloid from* T*.* crispa*, columbamine, showed strong acetyl-cholinesterase inhibitory activity with IC_50_ 48.10 *μ*mol/L. The structure-activity relationships derived from these results suggested that the quaternary nitrogen in the skeleton has some effect but that a high degree of methoxylation is more important for acetylcholinesterase inhibition [86].

*T*.* cordifolia *ethanol extract exhibited significant neuroprotection by increasing the dopamine levels (1.96 ± 0.20 and 2.45 ± 0.40 ng/mg of protein) and complex I activity (77.14 ± 0.89 and 78.50 ± 0.96 nmol/min/mg of protein) at 200 and 400 mg/kg, respectively [87]. Combination of ethanolic extracts of* T*.* cordifolia*,* B*.* monnieri,* and* E*.* alsinoides* in equal proportion provided synergistic nootropic effect on scopolamine induced amnesia in rats [88].

## 4. Chemical Constituents

**Figure 1:**
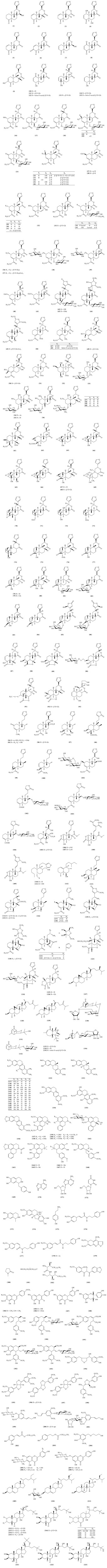
The structures of compounds isolated from* Tinospora*.

The chemical constituents of* Tinospora *have long been the pursued objectives of the researchers, and multiple classes of chemical constituents have been isolated and reported. Within these compounds, diterpenoids are the most dominant constituent found in the currently studied species in this genus. Up to now, 223 compounds have been isolated from genus* Tinospora*, including terpenoids, alkaloids, steroids, C_6_-C_3_ derivatives, and polysaccharides as well as other components. The compounds list and their structures are shown in Table 2 and Figure 1.

### 4.1. Terpenoids

#### 4.1.1. Diterpenes

Diterpenoids with clerodane-type skeleton represent the second most prevalent chemical class of the genus with over one hundred. They are the characteristic-hot constituents containing 20-C atom forming a 6/6 bicyclic skeleton. According to different stereochemistry of the decalin ring junction, this type of structure is grouped into* neo*-clerodane,* ent*-clerodane, and* nor*-*neo*-clerodane. The skeleton of* neo*-clerodane and* ent*-clerodane-type diterpene is the same, with some differences in the configuration. The spatial locations of angular methyl group linked at C-5, C-8, and C-9 are differed. Specifically, it is C-5(S), C-8(R), and C-9(S) for* neo*-clerodane and C-5(R), C-8(S), and C-9(R) for* ent*-clerodane.* Nor*-*neo*-clerodane is an oxidation product of* neo*-clerodane, and one more five-membered unsaturated lactone ring is added compared with* neo*-clerodane [89]. We prefer to divide the diterpenoids into another two major types. A naphthalene ring fused to a lactone ring forms the main basic structure of the diterpenoids. The C-9 position often has a six-membered side chain and usually forms a furan-ring moiety. Lactone ring commonly exists in this type of compounds. Most of the lactone rings connect between C-8 and C-12 constituting the three six-membered rings with the similar arrangement of phenanthrene. Meanwhile, those ones without a lactone ring linked at C-8 and C-12 account for another small part of terpenoids. Apart from what is mentioned above, the few ones with isomerization in ring C then are in another group.

*(1) Diterpenes with Type 1 Skeleton*. Compounds with type 1 skeleton are abundant in this genus. Due to the decalin core motif, Diels-Alder cycloaddition proved to be a valuable tool in their construction [90]. However, different synthetic routes result in the different positions of double bond and distinct substituents of the parallelized 6/6 bicyclic skeleton. These compounds are mostly reported from* T*.* cordifolia*,* T*.* crispa*, and* T*.* sagittata*, while other plant species possess a few. Compounds** 1**–**60** are typical clerodane-type diterpenes characterized by the presence of one or more double bond or methyl metabolites attached to the clerodane core [91–105]. Compounds** 61**–**86** have distinct connecting mode of lactone ring linked at C-1/C-18 with different spatial configurations of substituent groups [91–93, 106–112]. Compounds** 87**–**92**, as a tiny type, caused more attention to be paid to the ethane thiolate group [101, 108, 113, 114]. Tinosporaclerodanoid (**93**) is a particular clerodane-type diterpene without a furan-ring at C-12 superseded by a butane terminated glycol, never isolated from a natural or synthetic source before. Location of the carbonyl group is determined to be at C-1 [102].

*(2) Diterpenes with Type 2 Skeleton*. This is another type of clerodane diterpene without the C-8/C-12 linking lactone ring, yielding a variant genre of diterpenes featured by the decalin ring junction of different stereochemistry. In these compounds, the C-8 position always contains a methyl or methoxycarbonyl group, which obstructs the formation of the lactone bridge between C-8 and C-12; see compounds** 94** to** 112**. They are mainly identified from* T*.* cordifolia*,* T. tuberculate*,* T. sagittata *var*. yunnanensis*, and* T*.* sagittata*. There is an ester bond connected between C-1 and C-4 (**106**–**108**). One more sugar glucose unit in sagittatayunnanoside C (**103**) indicates that it may be the derivative of sagittatayunnanoside A (**102**) via the glycosylation of hydroxyl at C-18 [97, 115].

*(3) Diterpenes with Other Structures*. Compounds** 113**–**116** are structurally similar, and the differences between the basic structures are the oxidation degree of ring A. The diversity among their lactone ring may be an influencing factor causing these compounds to exhibit varying degrees of biological activities against the same cancer cell line [96, 115, 116]. Compounds** 117**–**120** were isolated from the stem of* T*.* rumphii*, and the lactone ring linking between C-6 and C-8 is quite remarkable [117, 118]. Compounds** 121**-**122** from* T*.* crispa* have the same nucleus structure as compound** 117**'s. Compounds** 123**–**126** are rearranged clerodane diterpene, obtained from the stems of* T*.* baenzigeri*, of which baenzigeride (**123**) and baenzigeroside (**124**), as the novel skeleton, arising from the* cis*-*ent*-*neo*-clerodane epoxide are considered to be the precursor of compounds** 125** and** 126 **[119].

#### 4.1.2. Other Terpenes

There are ten compounds in this category, involving three triterpenoids (**127**–**129**), six sesquiterpenoids (**130**–**135**), and one monoterpenoid (**136**). Cycloeuphordenol is a novel 31-nortriterpene in* T*.* malabarica*. The other two new triterpenes are cycloeucalenol and cycloeucalenone producing mild cardiotonic effects [120]. A new rearranged cadinane sesquiterpene glycoside, tinosinenside, is tested to have inhibitory activities against* R*-glucosidase [101]. Tinocordiside, consisting of a tricyclic skeleton with a cyclobutane ring, is found in the aqueous fraction of* T*.* cordifolia* [61, 121]. Compounds** 133**–**135** possessing the core structure of the parallelized five-membered and seven-membered rings are new daucane-type sesquiterpenes [122]. Compound** 136** is the only monoterpenoid isolated from the methanol extract of* T*.* cordifolia* [94].

### 4.2. Alkaloids

#### 4.2.1. Berberine Alkaloids

Twenty berberine alkaloids from genus* Tinospora* have been reported (**137**–**156**). Commonly, the exact positions of methoxy groups are linked at C-2, C-3, C-9, and C-10. Rings C and D are coplanar with each other and in good conjugation, yet rings A and B are not in the same plane because of the saturated carbons at C-5 and C-6. If the methyl linked at C-2 or C-3 is lost, it cannot be well stabilized by the vicinal unsaturated ring C. When the methyl is lost from C-9, a stable intermediate could be formed. After the loss of two H atoms at C-5 and C-6 (rings A and B are in a plane at this time), the two methoxy groups at C-2 and C-3 can also turn into a methylenedioxy group [139]. The polarity of the overall compound interrelates with the inhibitory of cholinesterases [86].

#### 4.2.2. Aporphine Alkaloids

Nine (**157**–**165**) aporphine alkaloids are isolated and characterized in this genus. When the hydroxyl is at C-1, it seems more favorable that the H_2_O-group tends to be lost in successive fragmentation. Possessing two pairs of vicinal hydroxyl and methoxy groups (usually between C-1 and C-2 and C-10 and C-11), two molecules of methanol moieties tend to be lost consecutively. In mass spectrometry, the losses of methyls and the second molecule of methanol may as well be considered to be minor fragmentation. Such changes may be related to the steric interactions between the hydroxyl at C-1 and the epoxy group at C-10 and C-11. Meanwhile, the stable aromatic ring prevents diversifications in the core structure [139].

#### 4.2.3. Other Alkaloids

Compounds** 166**–**169** are benzylisoquinoline alkaloids which play a significant role as a minute part. Compounds** 170**–**174** from* T*.* crispa* acted in concert on the cardiovascular system of anesthetized rats [142, 143]. Compounds** 175**–**178** are homologous provided with identical basic skeleton [149, 150].

### 4.3. C_6_-C_3_ and C_6_-C_3_-C_6_ Constituents

Other components isolated from the genus include six flavonoids, ten lignans, and several others [94, 103, 140]. Compounds** 183**–**188** belonging to flavonoid are the antioxidant profile of genus* Tinospora*. It is noteworthy that (−) epicatechin was reported for the first time in the stem extract of* T*.* cordifolia *[145]. Compounds** 189**–**198** are lignan in which** 192**–**194** were tested having obvious antioxidant activity [112]. Syringin (**199**) isolated from the crude butanol extract of* T*.* crispa* acted in concert with other active ingredients on the cardiovascular system [142]. Tinoscorside D (**200**) was isolated and elucidated based on spectroscopic data from the methanol extract of* T*.* cordifolia* aerial parts [94].

### 4.4. Steroids

*Tinospora* plants also contain steroids that are usually encountered in natural sources, and those presented in genus* Tinospora* are mostly derivatives of *β*-sitosterol. Up to now, fifteen of this kind of ingredients have been isolated and identified. Among them, *β*-sitosterol and *β*-sitosteryl glucoside were frequently found in* Tinospora* species [110, 113, 139, 144].

### 4.5. Polysaccharides

In the last decades polysaccharides from genus* Tinospora* have caught a great deal of attention due to their broad spectrum of therapeutic properties and relatively low toxicity, particularly being immune-enhancing agents. The composition of polysaccharides from* T*.* cordifolia* was reported as follows: glucose 98.0%, arabinose 0.5%, xylose 0.8%, galactose 0.3%, rhamnose 0.2%, and mannose 0.2% [151, 152]. Through subjecting to GC-MS analysis, the types of monosaccharides' linkages were investigated to be terminal-glucose, 4-xylose, 4-glucose, 4,6-glucose, and 2,3,4,6-glucose [153]. Sharma et al. reported that the active polysaccharide fractions mainly constitute glucose, fructose, and arabinose as monomer units. The polymorphonuclear leukocyte function test concluded that they were responsible for immune enhancement [135]. Administration of the polysaccharide fraction from* T*.* cordifolia* with tumor challenge inhibited (72.0%) the metastases formation in the lungs of syngeneic C57BL/6 mice [154]. Bioassay guided fractionation and separation of the ethanolic extract of* T*.* crispa* leaf led to four acylated glycosylflavonoids. Among the isolated compounds, isovitexin 2′′-(*E*)-*p*-coumarate (**206**) showed the best activity against *α*-glucosidase with an IC_50_ value of 4.3 ± 1.4 *μ*M [146].

## 5. Metabolism and Pharmacokinetics

To date, there are few pharmacokinetics studies of the extracts of this genus. Previous pharmacokinetics studies of* Tinospora* mainly focused on its diterpenes and its derived compounds that fitted to a one-compartment model. Shi et al. reported that columbin (**75**) and its derivatives (**72**,** 74**,** 80**, and** 83**) are commonly present in the root of* T*.* capillipes *and* T*.* sagittata*. Columbin is the major urinary metabolites in rats to which these compounds were orally given. These components could be absorbed and metabolized by the gastrointestinal tract. But pharmacokinetic studies showed that absorption of diterpene components was poorly absorbed through the gastrointestinal tract and they were quickly eliminated. Owing to the similar structures, these compounds have parallel pharmacokinetic parameters* in vivo*. Columbin is eliminated with systemic clearance of 5.04 ± 2.92 L/h/kg after i.v. administration demonstrating a little higher blood flow rate (3.3 L/h/kg in rats) than the hepar. It could be concluded that columbin was rapidly cleared via hepatic clearance and extrahepatic clearance. The volume of distribution at terminal phase was 8.73 ± 3.11 L/kg demonstrating a much higher total body water of 0.67 L/kg. The above researches suggested that columbin has extensive distribution into extravascular systems. Because columbin is almost insoluble in water, the absolute oral bioavailability of columbin is only 3.18 ± 2.22%. The time to reach the maximum plasma drug concentration is less than 5 mins. The aforementioned indicate that the absorption of columbin is limited and quickly eliminated [107, 155, 156]. So it is necessary to improve its absorption* in vivo* for clinical application in further research on pharmaceutical preparation. Since few pharmacokinetics studies of the extracts of* Tinospora* species have been reported, the needs for mechanism of actions on them are necessary in view of the wide bioactivities and extensive uses, and metabolism and pharmacokinetics studies are warranted to find out its most perspective activities.

## 6. Safety Evaluation

The clinical existence for herbs of genus* Tinospora* over centuries are in a way themselves evidences of therapeutic utility. No adverse effects have been reported in this genus prescribed under reasonable doses application.* T*.* cordifolia* is advocated as a tonic in infants and children to facilitate growth. The LD_50_ value for* T*.* cordifolia* is higher than 1 g/kg for oral administration [157]. Acute toxicity study with the dose of 3 g/kg demonstrated that* T*.* cordifolia* does not have any side effects and reported no death of the experimental rats [158]. When administered in doses of 0.1 g/kg for 12 weeks,* T*.* cordifolia* does not trigger any unfavorable factors on liver and renal function parameters in rats. It precipitated the increment of leukocytosis with neutrophilia in rats while no such effect was observed in healthy humans [27, 159].* T*.* cordifolia* treatment does not display clastogenicity and DNA damaging effect in bone marrow erythrocytes and peripheral blood lymphocytes [160]. And no neurological impairment or marked central nervous system depressant activities were shown [161]. Administration of* T*.* cordifolia* to healthy volunteers has been found to be safe in phase I study, and the drug was well tolerated [157, 162]. It has also been shown not to exert any conspicuous adverse reactions on the gastrointestinal system, renal system, cardiovascular system, and central nervous system [163]. However, systematic toxicological tests and safety evaluations have not been carried out extensively within the* Tinospora* species. Generally speaking, it could be used safely at low and middle dosage. Since test for toxicology of chemical constituents in this genus are rare until now, more toxicity researches remain to be done.

## 7. Conclusions

The review paper summarized a total of 223 compounds and abundant clerodane-type diterpene components that were reported from the genus* Tinospora*, with 152 references cited. We noted that* Tinospora* has an extensive distribution, targeted clinical application, and diverse biological and pharmacological activities of pure compounds and extracts. Previous phytochemical researches on the genus revealed the extensive presence of terpenoid, alkaloid, C_6_-C_3_ derivative, polysaccharide, and other compound types. The pharmacological activities were mainly regarded on antidiabetic, antioxidant, antitumor, anti-inflammatory, antimicrobial, antiosteoporosis, and immunostimulation activities. From the review, it can be seen that phytochemical investigations, pharmacological researches, and traditional clinical application mainly focus on 4* Tinospora* species,* T*.* sagittata*,* T*.* capillipes*,* T*.* cordifolia*, and* T*.* crispa*. The related chemical and biological activities toward other* Tinospora* species are still blank. So, plenty of further studies are necessary in order to illustrate the chemodiversity and to make full use of the biological significance of the compounds and extracts of* Tinospora*, especially the antidiabetic and antioxidant activities. Future works will be focused on the following aspects: to use* Tinospora* species as chemopreventive and therapeutic agent for various diseases, or to use them as adjuncts to exiting drugs to the treatment of diabetes, or to discover important and interesting biologically active compounds from these herbs by bioactivity-guided isolation. The authors wish the review can provide valuable data for explorations and advanced researches of* Tinospora* species.

## Figures and Tables

**Table 1:** Classic prescriptions of genus* Tinospora* in traditional and clinical usages.

Species	Preparation name/formulation	Main compositions	Medicinal uses	References
*T. sinensis*	Decoction	*Liquidambar formosana *Hance *Speranskia tuberculata *(Bge.) Baill. *Angelica sinensis *(Oliv.) Diels *Clematis chinensis *Osbeck *Bombyx mori *Linnaeus *Cinnamomum cassia *Presl *Achyranthes bidentata *Bl. *Spatholobus suberectus *Dunn *Aconitum kusnezoffii *Reichb. *Atractylodes lancea *(Thunb.) *Asarum heterotropoides *Fr.* Schmidt *var.* mandshuricum *(Maxim.) Kitag.	Knee joint osteoarthritis	[14]

*T. sinensis*	Qutan Tongluo Tang (祛痰*通络汤*)	*Arca subcrenata *Lischke *Arisaema cum Bile* *Drynaria fortune *(Kunze) J. Sm. *Dipsacus asper *Wall. ex Henry *Angelica sinensis *(Oliv.) Diels *Chaenomeles speciosa *(Sweet) Nakai *Eucommia ulmoides *Oliv. *Paeonia lactiflora *Pall. *Corydalis yanhusuo *W. T. Wang *Angelica pubescens *Maxim. F. biserrata Shan et Yuan *Akebia quinata *(Thunb.) Decne.	Lumbar disc herniation	[15]

*T. sinensis*	Huoxue Shujin Xunxifang (活血*舒筋熏*洗方)	*Clematis chinensis *Osbeck *Sparganium stoloniferum *Buch.-Ham. *Curcuma phaeocaulis *Val. *Uncaria rhynchophylla *(Miq.) Miq. ex Havil. *Carthamus tinctorius *L. *Salvia miltiorrhiza *Bge. *Ligusticum chuanxiong *Hort. *Achyranthes bidentata *Bl. *Aconitum kusnezoffii *Reichb. *Aucklandia lappa *Decne.	Ankylosis	[16]

*T. sinensis*	Sisheng Tang (四生汤)	*Rheum palmatum *L. *Clematis chinensis *Osbeck *Speranskia tuberculata *(Bge.)* Baill*. *Chaenomeles speciosa *(Sweet) Nakai *Arisaema erubescens *(Wall.) Schott *Aconitum kusnezoffii *Reichb. *Aconitum carmichaelii *Debx.	Fracture	[17]

*T. sinensis*	Fumigation	*Lycopodium japonicum *Thunb. *Speranskia tuberculata *(Bge.) Baill. *Spatholobus suberectus *Dunn *Prunus persica *(L.) Batsch *Carthamus tinctorius *L. *Angelica sinensis (Oliv.) Diels Ligusticum chuanxiong *Hort.	Ankle fracture	[18]

*T. sinensis*	Decoction	—	Visceral leishmaniasis	[19]

*T. capillipes*	Jinguo Heji (金果合剂)	*Scrophularia ningpoensis *Hensl. *Platycodon grandiflorum *(Jacq.) A. DC. *Glycyrrhiza uralensis *Fisch. *Ophiopogon japonicas *(L.f.) Ker Gawl. *Apis cerana *Fabricius	Pharyngitis	[20]

*T. capillipes*	Soaking solution	—	Infusion phlebitis	[21]

*T. capillipes*	Powder	—	Upper respiratory tract infection Acute tonsillitis Laryngopharyngitis Acute gastroenteritis Pneumonia Tracheitis	[22]

*T. cordifolia*	Decoction	*Berberis aristata* *Terminalia chebula* *Zingiber officinale*	Experimental amoebic liver abscess	[29]

*T. cordifolia*	Decoction	*Phyllanthus niruri* *Terminalia belerica* *Terminalia chebula* *Phyllanthus emblica*	Carbon tetrachloride induced hepatotoxicity	[30]

*T. cordifolia*	Decoction	—	Diabetic foot ulcers	[31]

*T. cordifolia*	Decoction	—	Allergic rhinitis	[32]

*T. cordifolia*	Decoction	—	Obstructive jaundice, tuberculosis, sepsis, breast cancer	[27]

*T. cordifolia*	Decoction	*Boerhavia diffusa* *Berberis aristata* *Terminalia chebula* *Zingiber officinale*	*Entamoeba histolytica*	[33]

*T. cordifolia*	Decoction	*Zingiber officinale* *Withania somnifera* *Tribulus terrestris*	Rheumatoid arthritis	[34]

**Table 2:** The compound list of genus *Tinospora*.

Number	Compounds	Species (part of plant)	References
Terpenoids			
1	Tinocapillins C	*T. capillipes *(*R*)	[123]
2	Tinocallone A	*T*. *capillipes *(*R*)	[107]
3	Tinocallone B	*T*. *capillipes *(*R*)	[107]
4	Tinocordin	*T. cordifolia*	[124]
5	Tinocapillins A	*T. capillipes *(*R*)	[123]
6	Fibaruretin H	*T. sagittata *(*R*)	[125]
7	Tinocapillins B	*T. capillipes *(*R*)	[123]
8	Fibaruretin G	*T. sagittata *(*R*)	[125]
9	Sagitone	*T*. *sagittata *var. *yunnanensis *(*R*)	[93]
10	Tinosponone	*T*. *cordifolia *(*S*)	[113]
11	Tinosporaside	*T*. *cordifolia *(*S*)	[100, 113]
12	Tinosporaside tetraacetate	*T*. *cordifolia *(*S*)	[113]
13	Amritoside D	*T*. *cordifolia *(*S*)	[109]
14	Tinocordioside	*T*. *cordifolia *(*S*)	[109]
15	Tinocordioside tetraacetate	*T*. *cordifolia *(*S*)	[109]
16	Boropetol B	*T. crispa *(*S*)	[126]
17	Tinocrisposide	*T. crispa *(*S*)	[127]
18	Boropetoside F	*T. tuberculata *(*S*)	[128]
19	(2R,5R,6S,9S,10S,12S)-15,16-Epoxy-2-hydroxy-6-*O*-(*β*-D-glucopyranosyl)-cleroda-3,7,13(16),14-tetraen-17,12-olid-18-oic acid methyl ester	*T*. *crispa*	[114]
20	(5R,6S,9S,10S,12S)-15,16-Epoxy-2-oxo-6-*O*-(*β*-D-glucopyranosyl)-cleroda-3,7,13(16),14-tetraen-17,12-olid-18-oic acid methyl ester	*T*. *crispa*	[91]
21	(5R,6R,8S,9R,10S,12S)-15,16-Epoxy-2-oxo-6-*O*-(*β*-D-glucopyranosyl)-cleroda-3,13(16),14-trien-17,12-olid-18-oic acid methyl ester	*T*. *crispa*	[91]
22	(2R,5R,6R,8S,9S,10S,12S)-15,16-Epoxy-2-hydroxy-6-*O*-{*β*-D-glucopyranosyl-(1→6)-*α*-D-xylopyranosyl}-cleroda-3,13(16),14-trien-17,12-olid-18-oic acid methyl ester	*T*. *crispa*	[91]
23	(2R,5R,6R,8R,9S,10S,12S)-15,16-Epoxy-2-hydroxy-6-*O*-(*β*-D-glucopyranosyl)-cleroda-3,13(16),14-trien-17,12-olid-18-oic acid methyl ester	*T*. *crispa*	[91]
24	(5R,6R,8S,9R,10R,12S)-15,16-Epoxy-2-oxo-6-*O*-(*β*-D-glucopyranosyl)-cleroda-3,13(16),14-trien-17,12-olid-18-oic acid methyl ester	*T*. *crispa*	[91]
25	Borapetoside B	*T*. *crispa*	[38, 91]
26	Borapetoside C	*T*. *crispa*	[38, 91]
27	Tinophylloloside	*T*. *sagittata *var. *yunnanensis *(*R*)	[92]
28	Epitinophylloloside	*T*. *sagittata *var. *yunnanensis *(*R*) *T*. *capillipes *(*R*)	[107]
29	Tinoscorside C	*T*. *cordifolia*	[94]
30	Borapetoside F	*T*. *cordifolia*	[94]
31	Borapetoside B	*T*. *cordifolia*	[94]
32	Tinocrispol A	*T*. *crispa *(*S*)	[129]
33	Amritoside C	*T*. *cordifolia *(*S*)	[109]
34	2-*O*-Lactoylborapetoside B	*T*. *crispa *(*S*)	[129]
35	6′-*O*-Lactoylborapetoside B	*T*. *crispa *(*S*)	[129]
36	Borapetoside H	*T*. *tuberculata *(*S*)	[95]
37	Octa-*O*-acetylborapetoside H	*T*. *tuberculata *(*S*)	[95]
38	Borapetoside H	*T. crispa *(*S*)	[96]
39	Tinospinoside C	*T. sagittata *var*. yunnanensis *(*R*)	[97]
40	Tinospinoside A	*T. sagittata *(Oliv.)Gagnep.(*R*)	[98]
41	Tinospinoside B	*T. sagittata *(Oliv.)Gagnep.(*R*)	[98]
42	Rumphioside A	*T*. *rumphii *(*S*)	[117]
43	Rumphioside B	*T*. *rumphii *(*S*)	[117]
44	Rumphioside C	*T*. *rumphii *(*S*)	[117]
45	Rumphioside D	*T*. *rumphii *(*S*)	[117]
46	Furanoid diterpene glycoside	*T. cordifolia *(*S*)	[130]
47	Cordioside	*T*. *cordifolia *(*S*)	[99]
48	Cordioside tetraacetate	*T*. *cordifolia *(*S*)	[99]
49	Cordifoliside D	*T*. *cordifolia *(*S*)	[100]
50	Cordifoliside E	*T*. *cordifolia *(*S*)	[100]
51	Boropetoside G	*T. tuberculata *(*S*)	[128]
52	Malabarolide	*T. malabarica *(*S*)	[131]
53	Tinosposinenside A	*T*. *sinensis *(*S*)	[101]
54	Tinosposinenside B	*T*. *sinensis *(*S*)	[101]
55	Tinosposinenside C	*T*. *sinensis *(*S*)	[101]
56	1-Deacetyltinosposide A	*T*. *sinensis *(*S*)	[103]
57	Tinosineside A	*T*. *sinensis *(*S*)	[103, 104]
58	Tinosineside B	*T*. *sinensis *(*S*)	[104]
59	Penta-*O*-acetyl-tinosineside A	*T*. *sinensis *(*S*)	[104]
60	Hexa-*O*-acetyl-tinosineside A	*T*. *sinensis *(*S*)	[104]
61	(3R,4R,5R,6S,8R,9S,10S,12S)-15,16-Epoxy-3,4-epoxy-6-*O*-(*β*-D-glucopyranosyl)-cleroda-3,13(16),14-trien-17,12-olid-18-oic acid methyl ester	*T*. *crispa*	[91]
62	Tinospinoside D	*T. sagittata *(*R*)	[105, 132]
63	Tinosporide	*T. cordifolia *(*S*)	[113]
64	Furanolactone clerodane diterpene	*T. cordifolia *(*S*)	[113]
65	Furanolactone clerodane diterpene	*T. cordifolia *(*S*)	[113]
66	Tinosporicide	*T. malabarica *(*S*)	[113]
67	Jateorin	*T*. *smilacina *(*S*)	[114]
68	Palmatoside F	*T*. *smilacina *(*S*)	[114]
69	(5R,10R)-4R,8R-Dihydroxy-2S,3R:15,16-diepoxycleroda-13(16),17,12S,18,1S-dilactone	*T*. *cordifolia *(*S*)	[110]
70	Menispermacide	*T. malabarica *(*S*)	[127]
71	Tinosporaside	*T. cordifolia *(*S*)	[113]
72	Ioa-hydroxy columbin	*T. malabarica *(*S*)	[131]
73	Tinoside	*T. capillipes *(*Rh*)	[107]
74	Isocolumbin	*T. capillipes *(*Rh*)	[107]
75	Columbin	*T*. *craveniana *(*R*) *T*. *sagittata *var. *yunnanensis *(*R*) *T*. *capillipes *(*R*)	[92, 93, 106, 107, 132, 133]
76	Palmatoside C	*T*. *sagittata *var. *yunnanensis *(*R*) *T*. *capillipes *(*R*)	[93, 107, 115]
77	Fibleucin	*T*. *sagittata *var. *yunnanensis *(*R*) *T*. *capillipes *(*R*)	[93]
78	Isocolumbin	*T*. *capillipes *(*R*) *T*. *sagittata *(*R*) *T*. *craveniana *(*R*)	[92, 106, 107, 133]
79	Tinoside	*T*. *sagittata *(*R*) *T*. *capillipes *(*R*)	[106]
80	6-Hydroxy columbin	*T*. *sagittata *(*R*)	[92]
81	(1R,4S,5R,8S,9R,10S,12S)-15,16-Epoxy-4-*O*-(*β*-D-glucopyranosyl)-cleroda-2,13(16),14-triene-17(12),18(1)-diolide	*T*. *crispa*	[91]
82	Tinospinoside E	*T. sagittata *var*. yunnanensis *(*R*)	[98]
83	8-Hydroxy columbin	*T. cordifolia*	[116, 128]
84	Tinospin E	*T. sagittata *(*R*)	[105, 112]
85	Cordifolide B	*T*. *cordifolia *(*S*)	[108]
86	Cordifolide C	*T*. *cordifolia *(*S*)	[108]
87	Furanolactone diterpene	*T. cordifolia *(*S*)	[113]
88	Boropetol A	*T. crispa *(*S*)	[134]
89	Boropetoside A	*T. crispa *(*S*)	[134]
90	Cordifolide A	*T*. *cordifolia *(*S*)	[108]
91	Menispermacide	*T*. *smilacina *(*S*)	[114]
92	Borapetoside A	*T*. *crispa*	[91]
93	Tinosporaclerodanoid	*T*. *cordifolia *(*S*)	[102]
94	Tinotufolin A	*T. tuberculata *(*L*)	[116, 128]
95	Tinotufolin B	*T. tuberculata *(*L*)	[116, 128]
96	Amritoside B	*T*. *cordifolia *(*S*)	[109]
97	Tinotufolin C	*T. crispa *(*S*)	[96]
98	Methyl(1*α*,4*α*a,5*α*,6*β*,8a*α*)-5-2-(3-furan-3-ene-2-one)ethyl-1,2,3,4,4a,5,6,7,8,8a-decahydro-1,2-dihydroxy-1-naphthalenecarboxylate	*T*. *rumphii *(*L*)	[115]
99	Tinotufolin F	*T. crispa *(*S*)	[96]
100	Tinotufolin E	*T. crispa *(*S*)	[96]
101	Sagittatayunnanoside B	*T. sagittata *var*. yunnanensis *(*R*)	[97]
102	Sagittatayunnanoside A	*T. sagittata *var*. yunnanensis *(*R*)	[97]
103	Sagittatayunnanoside C	*T. sagittata *var*. yunnanensis *(*R*)	[97]
104	Sagittatayunnanoside D	*T. sagittata *var*. yunnanensis *(*R*)	[97]
105	Amritoside A	*T*. *cordifolia *(*S*)	[109]
106	Tinosagittone A	*T*. *sagittata *(*R*)	[92]
107	Tinosagittone B	*T*. *sagittata *(*R*)	[92]
108	Tinocapilactone A	*T*. *capillipes*	[107]
109	Tinocapilactone B	*T*. *capillipes*	[107]
110	Tinosporafuranol	*T*. *cordifolia *(*S*)	[102]
111	Tinosporaclerodanol	*T*. *cordifolia *(*S*)	[102]
112	Tinosporafurandiol	*T*. *cordifolia *(*S*)	[100]
113	(2a*β*,3*α*,5a*β*,6*β*,7*α*,8a*α*)-6-2-(3-Furanyl)ethyl-2a,3,4,5,5a,6,7,8,8a,8b-decahydro-2a,3-dihydroxy-6,7,8b-trimethyl-2H-naphtho1,8-*bc*furan-2-one	*T*. *rumphii *(*L*)	[104–115]
114	Boropetoside D	*T. tuberculata *(*S*)	[116, 128]
115	Boropetoside E	*T. tuberculata *(*S*)	[116, 128]
116	Tinotufolin D	*T. crispa *(*S*)	[96]
117	Borapetoside E	*T*. *rumphii *(*S*)	[118]
118	Rumphioside I	*T*. *rumphii *(*S*)	[118]
119	Rumphioside F	*T*. *rumphii *(*S*)	[117]
120	Rumphioside E	*T*. *rumphii *(*S*)	[117]
121	(2*R*,7*S*,8*S*)-8-[(2*S*)-2-(3,4-Dihydroxy-2,5-dimethoxytetrahydro-3-furanyl)-2-hydroxyethyl]-2,8-dimethyl-10-oxo-11-oxatricyclo[7.2.1.0^2,7^]dodec-3-ene-3-carboxylate	*T*. *crispa*	[91]
122	Borapetoside D	*T*. *crispa*	[91]
123	Baenzigeride B	*T*. *baenzigeri *(*S*)	[120]
124	Baenzigeroside B	*T*. *baenzigeri *(*S*)	[120]
125	Baenzigeride A	*T*. *baezigeri *(*S*)	[120]
126	Baenzigeroside A	*T*. *baezigeri *(*S*)	[120]
127	Cycloeuphordenol	*T. malabarica *(*S*)	[113]
128	Cycloeucalenol	*T*. *crispa *(*S*)	[120]
129	Cycloeucalenone	*T*. *crispa *(*S*)	[120]
130	Tinosinenside	*T*. *sinensis *(*S*)	[101]
131	11-Hydroxymustakone	*T*. *capillipes*	[61, 121, 135]
132	Tinocordiside	*T*. *capillipes*	[61, 121, 135]
133	Tinocordifolioside	*T*. *sinensis *(*S*) *T*. *cordifolia *(*S*)	[103, 122, 136]
134	Tinocordifolin	*T*. *cordifolia *(*S*)	[136]
135	Tinocordifolioside tetraacetate	*T*. *cordifolia *(*S*)	[136]
136	Angelicoidenol-2-*O*-*β*-D-apiofuranosyl-(1→6)-*β*-D-glucopyranoside	*T*. *cordifolia*	[94]

Alkaloids			
137	Berberine	*T. cordifolia *(*S*)	[113]
138	Palmatine	*T. cordifolia *(*S*) *T. malabarica *(*S*)	[127, 137]
139	Jatrorrhizine	*T. cordifolia *(*S*) *T. capillipes *(*R*)	[137, 138]
140	13-Hydroxycolumbamine	*T*. *sagittata *(*R*) *T*. *capillipes *(*R*)	[14, 139]
141	13-Hydroxyjatrorrhizine	*T*. *sagittata *(*R*) *T*. *capillipes *(*R*)	[14, 139]
142	Demethyleneberberine	*T*. *sagittata *(*R*) *T*. *capillipes *(*R*)	[14, 139]
143	13-Hydroxypalmatine	*T*. *sagittata *(*R*) *T*. *capillipes *(*R*)	[14, 139]
144	Columbamine	*T*. *sagittata *(*R*) *T*. *capillipes *(*R*)	[14, 139]
145	Jatrorrhizine	*T*. *sagittata *(*R*) *T*. *capillipes *(*R*) *T*. *sinensis *(*S*)	[14, 106, 139, 140]
146	Isocolumbamine	*T*. *hainanensis *(*S*) *T*. *capillipes *(*R*) *T*. *sagittata *(*R*)	[106]
147	Stepharanine	*T*. *sagittata *(*R*) *T*. *capillipes *(*R*)	[14, 139]
148	Dehydrodiscretamine	*T*. *sagittata *(*R*) *T*. *capillipes *(*R*)	[14, 139]
149	Dehydrocorydalmine	*T*. *sagittata *(*R*) *T*. *capillipes *(*R*)	[14, 106, 139]
150	Palmaturbine	*T*. *sinensis* *T*. *craveniana *(*R*)	[14, 133, 139, 140]
151	Palmatine	*T*. *sinensis *(*S*) *T*. *capillipes *(*R*) *T*. *craveniana *(*R*)	[103, 106, 133, 140]
152	S-*trans*-N-Methyltetrahydrocolumbamine	*T*. *hainanensis *(*S*)	[141]
153	S-*trans*-Cyclanoline	*T*. *hainanensis *(*S*)	[141]
154	3-Hydroxy-2,9,11-trimethoxy-5,6-dihydro-isoquino3,2-a-isoquinolinylium	*T*. *sinensis*	[140]
155	4,13-Dihydroxy-2,8,9-trimethoxydibenzo [a,g] quinolizinium	*T. crispa*	[86]
156	Berberine	*T*. *smilacina *(*S*)	[92]
157	Magnoflorine	*T. cordifolia *(*S*) *T. malabarica *(*S*) *T. capillipes *(*R*)	[113, 138]
158	Menisperine	*T. capillipes *(*R*) *T. cordifolia *(*S*)	[113, 138]
159	N-Formyl anonaine	*T. crispa *(*S*) *T. malabarica *(*S*)	[113]
160	N-Formyl nornuciferine	*T. crispa *(*S*)	[113]
161	N-Acetyl nornuciferine	*T. crispa *(*S*)	[38]
162	N-Formylasimilobine-2-*O*-*β*-D-glucopyranoside	*T. crispa *(*S*)	[96]
163	N-Formylannonain	*T*. *capillipes*	[61, 135]
164	Magnoflorine	*T*. *sagittata *(*R*) *T*. *capillipes *(*R*)	[14, 106, 139]
165	Menisperine	*T*. *sagittata *(*R*) *T*. *capillipes *(*R*)	[14, 139]
166	Tembetarine	*T*. *sagittata *(*R*) *T*. *capillipes *(*R*)	[14, 139]
167	Reticuline	*T*. *sagittata *(*R*) *T*. *capillipes *(*R*)	[14, 139]
168	Tembetarine	*T. cordifolia *(*S*)	[113]
169	Higenamine	*T. crispa*	[142]
170	Adenine	*T. crispa*	[142]
171	Adenosine	*T. crispa*	[143]
172	Uridine	*T. crispa*	[142]
173	Salsolinol	*T. crispa*	[142]
174	(−)-Litcubinine	*T. crispa*	[142]
175	Tyramine	*T. crispa*	[142]
176	N-*trans*-Feruloyl tyramine	*T. tuberdata *(*S*)	[38]
177	N-*cis*-Feruloyl tyramine	*T. tuberculata *(*S*)	[36–38]
178	N-*trans*-Feruloyl tyramine diacetate	*T*. *cordifolia *(*S*)	[38, 136]
179	Kokusaginine	*T. malabarica *(*S*)	[113]
180	N-Methyl-2-pyrrolidone	*T*. *capillipes*	[61, 135]
181	Choline	*T. cordifolia *(*S*)	[137]
182	2,3-Dihydroxy-N-(2S,3S,4R)-1,3,4-trihydroxyicosan-2-yltetracosanamide	*T. oblongifolia*	[144]

C_6_-C_3_-C_6_			
183	Tinosporinone	*T. malabarica *(*HW*)	[113]
184	5-Alloxy-6,7,4′-trimethyl flavone	*T. malabarica *(*HW*)	[113]
185	Kaempferol	*T. malabarica *(*S*)	[113]
186	Astragalin	*T. malabarica *(*S*)	[113]
187	Quercitroside	*T. malabarica *(*S*)	[113]
188	(−)-Epicatechin	*T. cordifolia *(*S*)	[145]
189	Ecoisolariciresinol	*T. crispa*	[38]
190	Secoisolariciresinol-9′-*O*-*β*-D-glucopyranoside	*T*. *capillipes*	[38, 94]
191	Sagitiside A	*T. sagittata *var*. yunnanensis *(*R*)	[105, 112]
192	(+)-Lyoniresinol-2*α*-*O*-*β*-D-glucopyranoside	*T. sagittata *var*. yunnanensis *(*R*)	[105, 112]
193	(+)-5′-Methoxyisolariciresinol-3*α*-*O*-*β*-D-glucopyranoside	*T. sagittata *var*. yunnanensis *(*R*)	[105, 112]
194	3(a,4-dihydroxy-3-methoxybenzyl)-4-(4-hydroxy-3-methoxybenzyl) tetrahydrofuran	*T. cordifolia *(*S*)	[113]
195	Lirioresino-*β*-dimethyl ether	*T*. *sinensis*	[140]
196	Pinoresinol-di-*O*-glucoside	*T*. *capillipes*	[94]
197	(−)-Pinoresinol-4-*O*-*β*-D-glucopyranoside	*T*. *sinensis *(*S*)	[103]
198	8′-Epitanegool	*T*. *sinensis *(*S*)	[103]
199	Syringin	*T. crispa*	[142]
200	Tinoscorside D	*T*. *capillipes*	[94]
201	Cordifolioside A	*T*. *capillipes*	[38, 61, 135]
202	Docosyl-3,4-dihydroxy-*trans*-cinnamate	*T. oblongifolia*	[144]
203	2-(40-Hydroxyphenyl)-ethyl lignocerate	*T. oblongifolia*	[144]
204	Tinotuberide	*T. crispa *(*S*)	[113]
205	Isoorientin 2′′-*O*-(E)-sinapate	*T. crispa *(*L*)	[146]
206	2′′-(*E*)-*p*-coumarate	*T. crispa *(*L*)	[146]
207	Cosmosiin 6′′-(E)-ferulate	*T. crispa *(*L*)	[146]
208	Cosmosiin 6′′-(E)-cinnamate	*T. crispa *(*L*)	[146]

Steroids			
209	*β*-Sitosterol	*T. oblongifolia*	[144]
210	*β*-Sitosterol glycoside	*T. oblongifolia*	[144]
211	*β*-Sitosterol	*T*. *cordifolia *(*S*) *T*. *sinensis *(*S*) *T*. *craveniana *(*R*)	[102, 140]
212	2-Deoxy crustecdysone	*T. capillipes *(*R*)	[113]
213	2-Deoxy-3-epicrustecdysone	*T. capillipes *(*R*)	[113]
214	2-Deoxy-3-epicrustecdysone-3-*O*-D-glucopyranoside	*T. capillipes *(*R*)	[113]
215	20*α*-Hydroxy ecdysone	*T. cordifolia *(*S*)	[113]
216	20*β*-Hydroxy-ecdysone-2-*O*-*β*-glucopyranoside	*T*. *craveniana *(*R*)	[147]
217	20*β-*Hydroxyecdysone	*T*. *sagittata *(*R*) *T*. *capillipes *(*R*) *T*. *craveniana *(*R*)	[14, 106, 133, 139]
218	2-Deoxy-20*β-*hydroxyecdysone	*T*. *sagittata *(*R*) *T*. *capillipes *(*R*)	[14, 106, 139]
219	2-Deoxy-3-epi-20*β-*hydroxyecdysone	*T*. *sagittata *(*R*) *T*. *capillipes *(*R*)	[14, 139]
220	2-Deoxy-20*β-*hydroxyecdysone-3-*O*-glucopyranoside	*T*. *sagittata *(*R*) *T*. *capillipes *(*R*)	[14, 106, 139]
221	Polypodine B 20,22-acetonide	*T*. *cordifolia*	[94]
222	Makisterone A	*T*. *hainanensis *(*S*)	[148]
223	24-Epi-makisterone A	*T*. *hainanensis *(*S*)	[148]
